# Innovative practices in humanistic and liberal education: a four-in-one “infusive” educational model regarding character, literature, culture, and civilization

**DOI:** 10.3389/fpsyg.2026.1905343

**Published:** 2026-08-06

**Authors:** Yuanchun Liu, Mingzhao He, Jinmeng Dou

**Affiliations:** 1 Department of Chinese Language and Literature, School of Humanities, Shanghai Jiao Tong University, Shanghai, China; 2 High School Affiliated to Shanghai Jiao Tong University, Shanghai, China

**Keywords:** cultural transmission, higher education, humanistic and liberal education, ideological and political education, infusive education

## Abstract

**Introduction:**

This study addresses persistent challenges in China's humanistic and liberal education, including cultural disjunction, weakened value guidance, and insufficient integration of knowledge into practice. To overcome these limitations, this study proposes a four-in-one “infusive” educational model based on the interaction of character, literature, culture, and civilization.

**Methods:**

The model was developed through theoretical construction, literature synthesis, and pedagogical practice, and was empirically evaluated in the Chinese Character Culture course at Shanghai Jaio Tong University. A quasi-experimental design was employed, comparing an experimental group receiving infusive instruction with two control groups–a traditional multimedia teaching group and a no-course group. Data were collected through quantitative measures (*t*-tests and ANOVA) and qualitative evidence (interviews, course assignments, and follow-up investigations).

**Results:**

The findings provide empirical support for the feasibility and effectiveness of the proposed four-in-one educational model. Compared with the control groups, students in the experimental group demonstrated greater improvements in cultural self-awareness, value internalization, and overall learning satisfaction. They also exhibited stronger cultural identity, higher levels of engagement, and a greater ability to connect disciplinary knowledge with lived values.

**Discussion:**

This study proposes a novel four-in-one pedagogical paradigm that integrates digital and physical learning environments with the internal logic of Chinese culture. Unlike conventional approaches to liberal education, the model promotes the internalization of cultural values through experiential learning while providing a scalable and replicable framework for innovation in humanistic education.

## 1 Introduction

Over the past two to three decades, talent cultivation in domestic universities has generally prioritized knowledge and skill training, with conspicuous deficiencies in humanistic literacy development and value internalization. Although European and American universities deliver civic value education via general education curricula—such as the daily Pledge of Allegiance on U.S. campuses and Harvard University's general education reforms advocating public value concepts—young people still face issues including weakened sense of cultural belonging and ambiguous value cognition. Against this backdrop, the educational philosophy of Curriculum-based Ideological and Political Education emerged, whose core connotation lies in integrating value edification into coursework and realizing the internalization of values alongside knowledge acquisition (Shen, 2018). Proposed in Shanghai's educational reform in 2014, this model has been fully rolled out across all universities nationwide since 2020 under the Guidelines for the Construction of Curriculum-based Ideological and Political Education in Institutions of Higher Education.

Humanistic general education serves as the core vehicle for fostering cultural self-awareness and advancing value internalization. Compared with the developmental bottlenecks of general education in Europe and America, humanistic general education within China's framework of Curriculum-based Ideological and Political Education has formed an institutionalized educational system (Gao and Zong, 2017), gradually resolving compatibility conflicts between Western general education concepts and China's local higher education systems (Fu, 2015). Nevertheless, prevailing teaching models still struggle to efficiently fulfill educational objectives such as constructing cultural identity and emotional immersion. How to cultivate stable cultural identity and value internalization among students through humanistic courses has become a critical challenge demanding urgent solutions in university teaching reforms.

Drawing on long-term teaching practice, this study constructs a four-in-one immersive educational model framed as “Characters ⇌ Literature ⇌ Culture ⇌ Civilization”. It adopts outstanding traditional Chinese culture as the medium for immersive instruction and explores optimized implementation pathways for humanistic general education. A quasi-experimental design is adopted in this research, with three groups established: an experimental group receiving AI + Human Instruction (HI) immersive teaching, a control group taught via traditional multimedia lectures, and a baseline reference group with no enrollment in relevant humanistic courses. Based on theoretical reviews and identified research gaps, directional research hypotheses are proposed centered on three core psychological variables: cultural identity, value internalization, and learning satisfaction:

H1: After intervention, the experimental group will score significantly higher than both the traditional multimedia control group and the baseline reference group on post-test measurements of cultural identity, value internalization, and learning satisfaction.
H2: Significant differences exist in pre-post score gains across the three groups on the above three variables; the experimental group achieves the largest improvement magnitude, followed by the traditional multimedia control group, while the baseline reference group exhibits the smallest gain.
H3: Relative to the two control groups, students in the experimental group demonstrate deeper cognitive depth in assignments (higher SOLO levels), stronger cultural emotional resonance in interviews, and more solid cultural identity.

## 2 Literature review

Current humanistic general education in Chinese higher education is plagued by practical drawbacks including fragmented cultural cognition, insufficient emotional arousal, single pathways for cultivating cultural identity, and obstacles to the implementation of value internalization. This study develops the four-in-one immersive teaching model of “Characters ⇌ Literature ⇌ Culture ⇌ Civilization”, taking four core educational psychological variables—cognitive depth, emotional resonance, cultural identity, and value internalization—as its primary observation thread, and constructing intervention pathways supported by AI technology and the ICAP engagement theory. Three pervasive limitations characterize existing analogous humanistic intervention studies: first, systematic sorting of measurement tools and observation indicators for intervention effects is lacking; second, few studies simultaneously integrate the four educational carriers of characters, literature, culture, and civilization; third, a coherent transmission chain connecting external classroom activities to internal psychological value internalization remains unestablished.

This literature review centers on the core psychological variables and research hypotheses of this study. It systematically sorts measurement schemes, practical outcomes, and inherent limitations of existing research, elaborates layer by layer the innovations and improvements of the present study, and provides theoretical support for the immersive intervention model. Logical divisions of the four research sections are as follows: cultural awareness constitutes the theoretical foundation for cultivating cultural identity and emotional resonance; AI adaptive learning acts as the technical carrier for expanding cognitive depth; the ICAP framework furnishes a basis for observing and testing students' in-class cognitive engagement; value internalization represents the ultimate psychological objective of this intervention model.

### 2.1 Cultural awareness in humanistic and liberal education

Cultural awareness is the underlying foundation for shaping students' cultural emotional resonance and constructing stable cultural identity, as well as a core observation variable in humanistic general education. Existing research defines the formation of cultural awareness as encompassing four core elements: dialogue, cognition, culture, and global participation (Yurtsever and Özel, 2021). In empirical measurement, scales are widely used to quantify two outcomes—students' sense of cultural belonging and cross-cultural sensitivity—supplemented by qualitative evidence from interviews and reflective journals (Kaihlanen et al., 2019). Both theoretical knowledge and immersive practice can elevate cultural awareness (Hultsjö et al., 2019). Role-playing, cross-cultural communication, and extracurricular cultural practices strengthen students' cultural empathy and native cultural belonging (Byram, 2012; Tschannen et al., 2025), confirming the facilitating effect of situational involvement on emotional resonance and underpinning the core design of long-term cultural permeation via “immersion” adopted in this study, which distinguishes it from short-term sensory “immersive” experiences. Dewey's “learning by doing” theory cited by (Knoll 2022) further verifies that practical activities propel students from superficial cognition to profound cultural comprehension. Nevertheless, existing intervention studies on cultural awareness bear prominent flaws:

First, most studies deliver instruction relying solely on a single cultural carrier without integrating multi-layered resources of characters, literature, and civilization, leaving students prone to fragmented cultural cognition and unable to deepen cognitive depth (Yan, 2021).

Second, numerous university cultural integration teaching practices remain perfunctory. Current intervention protocols lack standardized implementation procedures, making it impossible to sustainably evoke stable emotional resonance or foster long-term cultural identity formation.

Third, prevailing measurement tools only capture static cultural awareness scores without differentiating the three psychological mechanisms of cognition, emotion, and identity, rendering them incapable of unpacking the pathways through which interventions take effect.

Corresponding to the above limitations, two key optimizations are implemented in this study: (a) A four-dimensional linked teaching carrier is constructed to progressively expand students' cognitive depth layer by layer; (b) A three-dimensional scale is developed to separately measure cultural self-awareness (identity), value internalization, and learning experience (motivation/self-efficacy). Combined with SOLO coding of assignments and qualitative interview data, multi-dimensional and multi-source data are deployed to observe psychological shifts.

### 2.2 Application of AI-enhanced learning experiences in humanities education

Digital technology serves as a vital tool for expanding students' cognitive depth and enriching immersive learning scenarios. Empirical evidence confirms that AI-generated instructional videos and adaptive resource push powered by large language models can boost learning retention rates, stimulate intrinsic learning motivation, and trigger deep learning (Xu et al., 2025; Acquah et al., 2025). Research in this field primarily adopts academic performance, learning motivation scales, and classroom behavioral observations as outcome indicators, focusing on measuring superficial learning outcomes while rarely linking interventions to deep psychological variables such as cultural emotion and identity. Yet an inherent paradox exists in technology-enabled humanistic teaching: highly simulated immersive technologies tend to produce “strong sensory immersion yet weak intellectual engagement”. Students merely receive superficial sensory stimulation, fail to establish profound connections between cultural symbols and civilizational contexts, cannot advance cognitive depth, and struggle to generate lasting cultural emotional resonance (Cao et al., 2024).

Three major gaps exist in current research integrating AI and humanistic education:

(a) Technology application remains confined to superficial instrumental functions such as resource delivery and video display; AI is not embedded into a complete cultural education chain, failing to collaboratively facilitate identity formation and value internalization.
(b) Existing interventions fail to distinguish “sensory immersive experience” from “psychological permeating immersion”, over-reliance on hardware simulation with insufficient long-term continuous cultural guidance.
(c) Measurement indicators only focus on learning efficiency without tracking long-term impacts of AI intervention on students' cultural emotion and cognitive levels.

This study restructures and optimizes the AI application model: integrating the DeepSeek large model, RAG retrieval technology, AI agents, and digital teacher courseware into the four-dimensional teaching system. AI only undertakes auxiliary cognitive functions including knowledge guidance and document retrieval, while two-thirds of class hours are reserved for humanistic seminars, group practices, and other human-centered immersive sessions. Meanwhile, SOLO hierarchical coding quantifies students' cognitive depth, and scales track emotional and identity changes, addressing the bias of existing research that prioritizes efficiency over psychological mechanisms.

It should be noted that this study strictly distinguishes two distinct concepts: infusive mode and immersive mode. The immersive mode focuses on external situational construction and immediate sensory experience, while the infusive mode centers on long-term cultural penetration and internal value internalization. Furthermore, immersion places greater emphasis on the combination of technology, interpersonal interaction, teaching aids, and value cultivation, whereas infusion takes situational engagement, emotional resonance, subtle edification, and value identification as its core. The former acts as the implementary means, while the latter also includes the educational objective.

### 2.3 Application of the ICAP framework in humanities education

The ICAP framework explains students' in-class cognitive engagement through four modes: passive, active, constructive, and interactive engagement, constituting a classic theoretical tool for observing cognitive depth. Extensive empirical studies verify that higher-order engagement modes significantly improve deep learning outcomes (Thurn et al., 2023; Sánchez et al., 2022; Uzun et al., 2025). Prior research mainly gauges ICAP engagement effects via classroom behavioral observation, post-class test scores, and task completion quality, limited to observable external classroom behaviors and unable to capture underlying internal psychological shifts such as emotional resonance and identity formation. Scholars also note boundary conditions for ICAP application: activity design must be flexibly adjusted according to teaching content and student characteristics rather than mechanically applied. The framework carries inherent limitations rooted in its overt behaviorist orientation: it only focuses on observable external learning behaviors and cannot account for implicit emotional cultivation and identity construction processes unique to humanistic education; reliance solely on ICAP external activities cannot achieve profound value internalization. Overall, deficiencies of existing ICAP research applied to humanistic education include:

(a) Engagement is elevated merely through isolated single classroom activities, lacking long-term progressive cultural inquiry design that sustains emotional arousal.
(b) Evaluation systems only attend to cognitive engagement behaviors without establishing correlations between behaviors and psychological outcomes such as cultural identity and value internalization.
(c) Few studies combine the ICAP framework with digital AI tools and multi-layered cultural carriers to implement comprehensive interventions.

This study embeds the four-tier ICAP engagement logic into classroom design, deploying AI adaptive tasks to raise the proportion of constructive and interactive engagement. Beyond behavioral observation, scales, assignment coding, and interviews are integrated to comprehensively measure cognitive depth, emotion, and identity, remedying the ICAP framework's neglect of internal psychological mechanisms and bridging the transmission pathway from external classroom activities to internal value internalization.

### 2.4 Cultivation of value internalization in humanistic education

Value internalization represents the ultimate psychological objective of humanistic general education, manifested chiefly as stable cultural beliefs and cultural responsibility nurtured incrementally through course case studies, group seminars, historical critical reflection, and other activities (Chi and Wylie, 2014; Wong, 2023). Language and writing function as cultural carriers bridging cultural cognition and identity, a conclusion providing theoretical support for the character tracing segment of this study. Traditional research in this field predominantly adopts moral judgment scales and value questionnaires to measure outcomes, offering macro-level assessments of value cognition without unpacking the mediating roles of emotional resonance and identity formation. Review of prevailing general education practices reveals four unavoidable bottlenecks in cultivating value internalization:

(a) Superficial value cultivation: Instruction stops at knowledge lecturing without inquiry activities capable of evoking emotional resonance, disconnecting cultural cognition from internal psychological beliefs (Liu, 2023).
(b) Flat cultural cognition: Students cannot perceive multi-dimensional cultural connotations embedded in writing; superficial reading fails to generate profound cultural empathy.
(c) Absence of practical carriers: Lecture-centered teaching lacks immersive practice scenarios to continuously reinforce identity formation.
(d) Unitary evaluation systems centered on written exam scores, without standardized measurements of multi-dimensional psychological indicators including cognitive depth, emotion, and cultural identity.

Humanistic general education is contracting both domestically and internationally (Liu, 2023; Parker and Sykes, 2024), indirectly demonstrating insufficient effectiveness of existing value cultivation intervention models. Synthesizing literature across the four thematic sections, three core research gaps are identified:

(a) Most existing interventions adopt only two to three carriers selected from characters, literature, culture, and civilization, lacking a complete four-dimensional integrated teaching scheme.
(b) AI-enabled humanistic teaching remains superficial technical integration, without deep alignment with psychological objectives such as cultural identity and value internalization.
(c) Measurement systems in prevailing intervention research are unitary, incapable of simultaneously observing multiple psychological mechanisms including cognitive depth, emotional resonance, and cultural identity, resulting in ambiguous explanations of intervention pathways.

Systematic improvements are developed in response to all the above research gaps:

(a) Theoretical framework: For the first time, integrate the four carriers of characters, literature, culture, and civilization, paired with AI tools and ICAP cognitive engagement theory to construct a complete immersive intervention chain.
(b) Mechanism interpretation: Establish a chained effect pathway centered on four psychological variables—cognitive depth, emotional resonance, cultural identity, and value internalization—to clearly explain how classroom activities shape internal psychology through intervention.
(c) Measurement system: Integrate multi-source data including pre-post scale testing, SOLO cognitive coding of assignments, and semi-structured interviews to provide concurrent quantitative and qualitative evidence for multiple psychological outcomes, addressing the flaws of single-dimensional measurement and ambiguous mechanistic explanations in prior research.

## 3 Theoretical framework of the four-in-one “infusive” model

The four-in-one immersive model proposed in this paper is not merely a theoretical framework tailored to policy-oriented education systems. Instead, it addresses the prevalent deficiencies in contemporary youth's cultural cognition and value internalization, and maximizes the educational potential of fine traditional Chinese culture. Rather than delivering direct moral or ideological indoctrination, the teaching intervention in this study takes outstanding traditional Chinese culture as the learning medium and triggers a continuous sequence of psychological changes through immersive classroom scenarios. First, digital and collaborative group classroom designs boost students' learning engagement and activate their intrinsic motivation for traditional cultural learning. In-depth cultural inquiry evokes students' emotional resonance with culture and gradually fosters a positive cultural identity. Sustained inquiry-based learning and positive learning feedback continuously enhance students' academic self-efficacy, shifting their cognition from superficial memorization to high-order integration and critical thinking. Ultimately, positive cultural emotions, solid cultural identity, and in-depth cognition jointly facilitate the internalization of cultural values at the individual psychological level. The three-dimensional measurement tool adopted in this study corresponds to three core psychological elements: cultural identity, value internalization, learning motivation and self-efficacy, which quantitatively test the intervention effects along this psychological pathway.

### 3.1 Definitions of core concepts

#### 3.1.1 “Infusive” education

**Figure 1:**
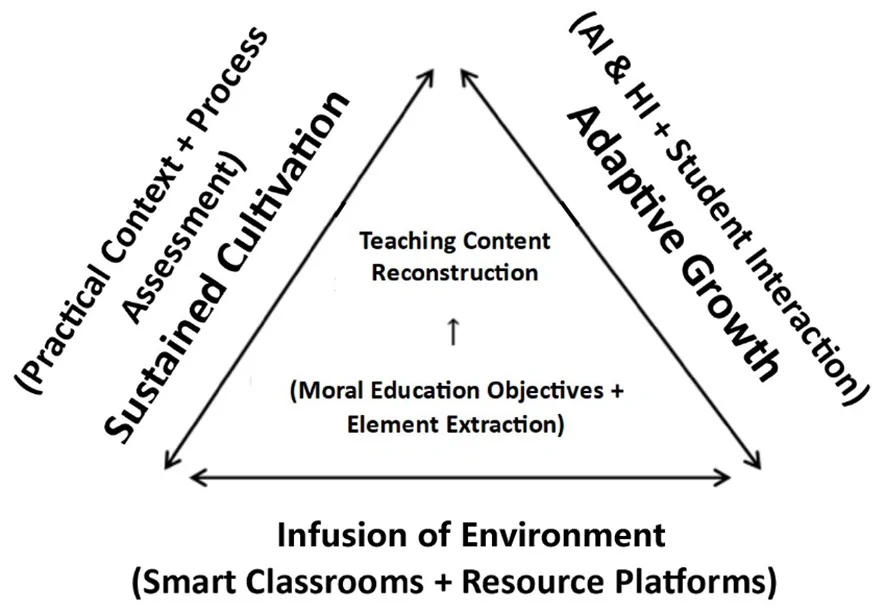
Pathway for the realization of the “infusive” educational model.

Immersive teaching in language and cultural learning centers on short-term situational engagement within second-language classrooms, adopts target languages as the medium of instruction, and realizes language acquisition via brief real-scene experiences (Johnson and Swain, 1997). International academia generally defines it as a short-term, scenario-restricted language acquisition method that prioritizes immediate sensory experience. This reference will also be added to the reference list. Nevertheless, the “infusive” educational model analyzed in this paper represents a pedagogical approach that subtly instills values, cultural connotations, and knowledge skills through comprehensive and multidimensional content, educational environments, and instructional methodologies. Focused on moral education, its essence lies in reconstructing teaching content while cultivating a sustained, holistic environment and experiential practice. This approach allows students to achieve knowledge construction and emotional development naturally, producing a gentle yet profound educational effect. The path to realization is illustrated in Figure 1.

#### 3.1.2 Relationship of the four-in-one model

“Character,” regarded as the cornerstone of cultural transmission, represents a key marker of the evolution of human civilization. Character encapsulates the transmission and documentation of thoughts, emotions, and history; for example, the oracle bone script character for “德 (morality)” reveals an early ethical perspective characterized by directness and righteousness. In this article, the character referenced encompasses linguistic characters and associated symbolic systems. Artistic expressions such as literature, music, and dance are considered extensions of linguistic characters, reflecting societal life and inner sentiments through creative expression. These cultural forms—from oracle bone scripts and bronze inscriptions to literary works, calligraphy, music, and dance—chronicle the civilizational journey of the Chinese nation. It fosters students' cultural emotional resonance and facilitates the construction of internal cultural identity through historical context, regional characteristics, ideological depth, and aesthetic values.

As a vessel of ancient and contemporary memory, “literature” encompasses various records inscribed in linguistic characters and related symbols, including classics, archives, oral histories, and artifacts. The literature provides material evidence of human development and embodies the transmission of material and spiritual culture, encompassing historical, artistic, and scientific values. A typical example is the anthropological framework known as the “Four-Pronged Evidence Approach” (Ye, 2010), which includes character literature, unearthed literature, oral and intangible cultural heritage, and visual and physical artifacts. Literature forms a vital foundation for studying history and culture, as it reflects social development from multiple perspectives. By engaging with literature, students can directly observe historical transformations, fostering a deeper understanding of diverse cultural characteristics and values while tracing the lineage of civilization.

The multifaceted construct of “culture,” as an embodiment of value orientation, encompasses viewpoints, beliefs, customs, arts, and technologies. The collective cultures of different ethnicities and regions form a treasure trove of human culture. Rather than engaging in indiscriminate indoctrination, liberal education should prioritize distinguishing between traditional culture and cultural traditions (Hao, 2021). By immersing themselves in exemplary traditional culture and multiculturalism, students broaden their horizons and cultivate respect for differences and enhance global perspectives and intercultural communication competencies, ultimately fostering cultural confidence. The successful localization of Marxism in China stems precisely from its integration with traditional Chinese culture—the unification of governance and morality and truth and goodness (Pang, 1993). Similarly, this principle applies to the interpretation of Chinese-style modernization.

“Civilization” signifies the hallmark of societal advancement and the achievements of material and spiritual domains. Character, literature, and culture constitute the core of civilization, driving its continuous development. From agrarian to information civilization, each advancement depends on the evolution of these elements. Humanistic and liberal education guide students in recognizing the trajectory of civilization, cultivating their sense of historical mission and social responsibility. Thus, students are encouraged to engage in technological innovation and cultural inheritance, contributing to the progress of human civilization. Practical situational immersion activities help internalize knowledge into values, reinforcing students' sense of responsibility.

Knowledge within the humanities is manifested through the interconnections among character, literature, culture, and civilization in this four-in-one framework. Character and literature, which bear and record significant information, form the foundation of culture and civilization. Literary arts transmit cultural values through characters, while literature preserves human memory and provides evidence for research. Culture imbues character and literature with soul and significance, while civilization represents a higher form of cultural development. These four elements are closely interconnected and mutually reinforcing, forming an organic and cohesive whole. Within the context of humanistic and liberal education, integrating these components establishes a comprehensive educational system that cultivates well-rounded individuals with solid cultural foundations, strong moral character, and a heightened sense of social responsibility. To leverage the joint force of the four elements, it is essential to continuously foster an infusive educational environment and implement practical teaching in an all-round manner.

### 3.2 Theoretical support

#### 3.2.1 SOLO taxonomy theory

The Structure of Observed Learning Outcome theory (SOLO; Biggs and Collis, 1982) categorizes learning outcomes into five hierarchical levels, ranging from low to high: Pre-structural, Uni-structural, Multi-structural, Relational, and Extended Abstract. Each level represents increasing depth and complexity in students' comprehension and application of knowledge. To assess learning progress and depth of understanding, humanities courses can design formative assessments based on the SOLO taxonomy. A typical example would be the curricular design related to classical Chinese poetry. Initially, multi-tiered tasks can be designed. Students at the “Uni-structural” level may recite or explain the fundamental content of an ancient poem. Conversely, those at the “Extended Abstract” level can analyze the historical context and cultural traditions, exploring the influence of ancient poetry on modern literature while articulating their insights. This design allows students to elevate their capabilities progressively according to their individual levels. After completing tasks at each level, specific feedback should be provided to help students recognize their progress and areas for improvement. Students should also be encouraged to reflect on their learning process, identify their position within the SOLO classification, and set subsequent learning goals. Through this process-oriented assessment, teachers can more accurately evaluate students' learning trajectories and offer targeted guidance, ultimately enhancing students' comprehension and application of humanities knowledge.

#### 3.2.2 ICAP framework

The ICAP framework distinguishes students' cognitive engagement during learning (Chi and Wylie, 2014). Interactive learning yields optimal outcomes, as students engage deeply through collaboration and discourse. Constructive learning requires learners to generate new knowledge. Active learning emphasizes student initiative in performing tasks. Conversely, passive learning involves merely receiving information. Based on this framework, patterns of student engagement, along with the levels and depth of knowledge construction, can be systematically analyzed. This analysis provides valuable insights for developing personalized instructional strategies and assessing learning effectiveness.

**Figure 2:**
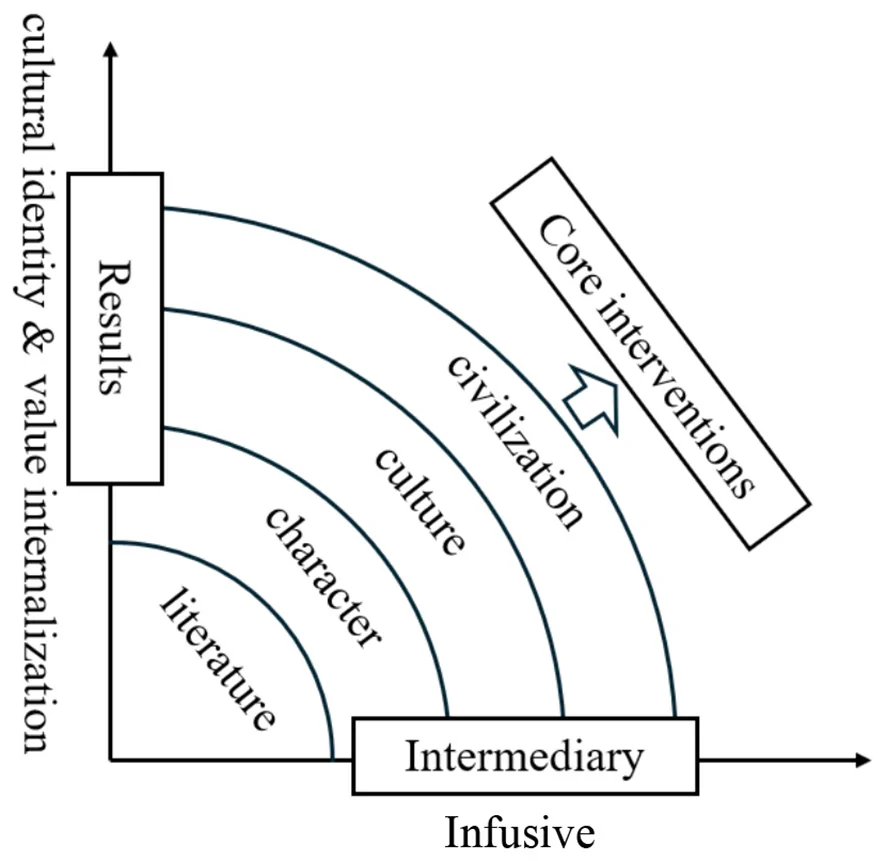
Theoretical model of the four-in-one infusive teaching model.

This study defines the four-in-one infusive model as a testable educational and teaching model that takes the four elements as the core intervention, uses infusive learning as the mediator, and regards value internalization and cultural identity as the outcomes. It aims to avoid the problem of purely conceptual interpretation found in previous studies. Specifically, the four elements are converted into observable and measurable indicators, including the accuracy rate of character cognition, practical completion level, literature analysis ability, cultural sensitivity, and civilization identity. The theoretical model is shown in Figure 2.

The proposal of this theoretical model aims to address the issues concerning cultural identity and value shaping. Therefore, in the subsequent empirical analysis, three key indicators directly relevant to these goals were measured: cultural self-awareness, the level of value internalization, and satisfaction with learning experience. The cultural self-awareness dimension measures students' sense of cultural belonging and the degree of their cultural identity cognition; the value internalization dimension gauges the extent to which students' external cultural perceptions transform into stable internal value beliefs; the learning experience satisfaction dimension reflects students' classroom engagement experience, learning self-confidence, and willingness to take initiative in learning. The content reconstruction and instructional design of the four elements (character, literature, culture, and civilization) serve as effective approaches to examine the above three key indicators.

#### 3.2.3 Core internationally relevant theories

(1) Experiential Learning Theory (Kolb, 1984).

(Kolb 1984) proposed a four-stage experiential cycle consisting of concrete experience, reflective observation, abstract conceptualization, and active experimentation. The theory posits that cognition originates from sustained contextual engagement, which differentiates it from short-term sensory immersive classrooms (Johnson and Swain, 1997, pp. 1–12). The cyclic practical design of “character tracing and cultural decoding” adopted in this study aligns with this theory. Sustained multi-layered cultural experiences facilitate continuous reflection, offering an international theoretical rationale for distinguishing long-term “permeating immersion” from brief “sensory immersion.”

(2) Cultural-Historical Activity Theory (Vygotsky, 1934/1987).

Vygotsky's cultural-historical activity theory states that higher psychological functions derive from social symbolic interactions mediated by cultural tools, and individual identity and value cognition are gradually internalized through group collaboration. This study takes Chinese characters and historical-literary documents as cultural symbolic carriers, and constructs social learning scenarios via group seminars and AI-assisted collaborative inquiry, explaining how characters and documents act as mediators to advance students' cultural psychological development.

(3) Transformative Learning Theory (Mezirow, 1997).

Mezirow's (1997) transformative learning theory argues that in-depth reflection can restructure individuals' original cognitive frameworks and bring about qualitative shifts in perspectives and internal beliefs. This model incorporates critical literature analysis and cultural reflection sessions to guide students to critically examine their inherent cultural perceptions, which corresponds to the formation pathway of the core research variable “value internalization.”

(4) Identity Formation Theory (Phinney and Ong, 2007).

Three-stage ethnic identity development model, proposed by (Phinney and Ong 2007), outlines a complete chain of cultural exploration, acceptance and identification, and stable identity construction, providing an international measurement reference for the indicator “cultural identity” in this study. The four-dimensional progressive design guides students to explore the lineage of characters and civilizations layer by layer, completing a psychological developmental process from superficial symbolic cognition to a stable sense of cultural belonging.

(5) Moral Development Theory (Kohlberg, 1984).

Kohlberg's (1984) stage theory of moral development distinguishes compliance with external rules from internal value identification and reveals the internalization logic of values shifting from external acceptance to inner conviction. Moving beyond mere moral judgment assessment, this study extends the theory to humanistic value cultivation, explaining how cultural learning enables students to form autonomous and stable cultural value beliefs.

### 3.3 Applicability explanation

The core framework of the infusive educational model constructed in this study can be transferred to courses in different disciplinary fields, possessing strong replicability and operability. Its underlying mechanism does not rely inherently on the single course of *Chinese Character Culture*. In future popularization, teachers can achieve optimal adaptation merely by replacing the core carriers of teaching content and designing corresponding instructional activities. For example, in general education courses on ancient Chinese literature, the carriers can be substituted with classic ancient Chinese texts and archival materials, and instructional design can be developed based on the historical information and embedded values conveyed in these literary documents. At the structural level, the model retains consistent implementation pathways, including infusive situational construction, compilation of cultural relic and documentary resources, and establishment of practical platforms, so as to maintain methodological coherence. Furthermore, this study stresses the importance of defining clear applicable boundaries for the model. We propose that it is preferentially suitable for courses oriented toward humanistic literacy cultivation, cultural inheritance, and value shaping, first and foremost humanities courses, followed by social science courses. By contrast, it is temporarily not applicable to skill-intensive, practice-based, and instrumental science and engineering courses. This delineation avoids the over-generalization and arbitrary application of the proposed model.

## 4 Practical pathways and empirical analysis

### 4.1 Course reconstruction and implementation

#### 4.1.1 Design of teaching content

The development of course-based ideological and political education, along with innovations in the teaching environment—such as the integration of artificial intelligence—should align with the fundamental objective of effective teaching. The four-in-one concept requires reconstructing teaching content by extracting moral education elements from the course.

(i) Extracting moral education elements and aligning talent orientation to nurture new generations. Humanities courses should be firmly grounded in the evolution of Chinese civilization and human civilization as a whole. They must follow the talent cultivation orientation of “value guidance, knowledge dissemination, capability development, and character formation.” Moreover, these courses should thoroughly explore the cultural connotations within their content and literature resources, meticulously extracting elements of moral education (see Table 1, “Points of Ideological and Political Integration”). Examples include patriotic spirit, national spirit, humanistic care, and social responsibility. Another specific example is the character education course. Its objectives include guiding students to understand the cultural connotations, national thought, and aesthetic spirit reflected in ancient scripts such as oracle bone scripts, bronze inscriptions, seal scripts, and clerical scripts. Elements of moral perspectives and worldviews intrinsic to Chinese characters should be distilled, facilitating a creative dialectical analysis of phenomena in Chinese character culture while safeguarding its purity and vitality. Through this process, students' positive cultural emotional resonance and perception of humanistic values are fostered. They are guided to inherit and promote China's rich traditional culture consciously, reinforcing awareness of the shared community of the Chinese nation. It is important to move beyond the narrow view of treating “humanities courses as ideological and political courses.” Professional knowledge should remain the core, while the integration of moral education is the overarching goal of student cultivation. Guided by Bloom's Taxonomy and cognitive theory, the first step is to set high-order affective goals. Each course's instructional design must delineate the ideological and political education elements in advance, with specific teaching segments structured to achieve these objectives.

**Table 1:** Comparison of course content before and after reconstruction.

Context (old version)	Reconstructed teaching content design			
	Chapter	Content	Cultural relics	Points of ideological and political integration
Origins of Chinese characters	I	Origins of Chinese characters and dawn of civilization	[Pottery]	Cherish their mother tongue
Oracle bone script	II	Oracle bone script and unity of man and nature	[Oracle bones]	The only ancient characters in use worldwide
Bronze inscriptions	III	Bronze inscriptions and the civilization of rites and music	[Bronze vessels]	Cultural tradition and spiritual essence of rites and music
Small and large seal scripts Scripts of six Eastern Kingdoms Clerical, cursive, running, and regular scripts Material characters since the warring states	IV	Seal script, scripts of six eastern Kingdoms, and continuous improvement through reforms	[Imperial seals] [Eaves tile]	Patriotism; The spirit of reform and innovation
	V	Han Dynasty clerical script and Tang Dynasty regular script and axial civilizations	[Bamboo slips] [Wei stele script]	The essence of harmony and the pursuit of Great Unity
Formation of characters	VI	Six categories of Chinese characters and national thinking	[Arrowheads]	Human-centered ideas; spirit of truth-seeking, goodness, and beauty
Standardization of scripts evasion of Taboo	VII	Chinese characters and political civilization	[Tiger tally] [Stone inscription of Qin Dynasty]	The worldview and moral concepts
Chinese characters and calligraphy Character games Chinese characters and literature	VIII	Chinese characters and national aesthetic spirit	[Calligraphy scrolls] [Thread-bound ancient books]	Eastern charm and poetic traditions,
Chinese characters and thoughts	IX	Chinese characters and traditional thoughts	[Portrait brick]	The significance of benevolence and valuing people-oriented thought
Dissemination of Chinese characters	X	Dissemination of Chinese characters and the unity of Chinese nations	[Overseas stone rubbings]	Chinese characters are symbols of cultural identity

(ii) Reconstructing teaching content, focusing on value guidance, and enhance students' cultural identity and sense of cultural belonging. Based on the distinct characteristics of different courses and teaching content, extracted moral education elements should be organically integrated into syllabi, teaching materials, and all aspects of teaching activities, constructing a systematic and comprehensive framework for course-based ideological and political education. An example is the “Chinese Character Culture” course at UNIVERSITY S. Following the thematic thread of “characters ⇌ cultural relics ⇌ culture ⇌ civilization,” the course integrates ideological and political elements into each session while reconstructing teaching content in alignment with cutting-edge academic disciplines. This method transforms the conventional model of similar courses, which typically focus solely on the fundamental knowledge of Chinese character culture. The reconstructed content exhibits greater dimensionality and smoother logic (see Table 1).

In the lesson on “Bronze Inscriptions and the Civilization of Rites and Music,” analysis focused on how bronze vessel inscriptions record King Wu's designation of Luoyi (an old name for Luoyang city) as the center of his dynasty, corresponding to the North Celestial Pole. The discussion explored the patriarchal clan system encapsulated in the term “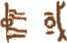.” Consequently, students were guided to contemplate the inherent centripetal force of Chinese civilization embodied in the “Relationship between Humans and Nature.” Students also considered values such as upholding a symbiotic relationship between humans and nature, fostering national identity, and contributing to the broader Chinese cultural sphere (Lu, 2023). This demonstrates that studying relevant cultural contexts and political systems facilitates students' comprehension of the civilizational consciousness and ethnic identity embedded in ancient characters. Thus, a transition from mere memorization of knowledge to a deeper understanding of culture can be achieved. Building on this, students were encouraged to interpret the significant implications of the two characters for future generations of the Chinese nation and the underlying reasons. In other words, the focus was on whether students could apply their acquired knowledge within relevant cultural contexts (Kiel and Linkov, 2025). Grounded in academic frontiers, this content reconstruction is more likely to foster a deep sense of patriotism among students, making the learning of moral education elements more resonant and impactful.

#### 4.1.2 Innovation in teaching methods

(Wu et al. 2025) proposed that “the technological pathways for empowering ideological education through human-machine collaboration can be realized from four aspects—data-driven information acquisition, virtual-physical integrated cognitive experiences, big data-driven knowledge discovery, and practice guidance coupled with theoretical iterations.” The four-in-one “infusive” model aims to leverage new technologies comprehensively to diversify teaching methodologies and evaluate effectiveness for continuous educational reform.

(i) Integration of AI and HI to construct “Four Intelligences” as one, stimulating innovative consciousness and cultivating humanistic sentiments. Courses in the humanities and liberal arts must remain aligned with contemporary developments. Grounded in the philosophy of “co-creation between teachers and students,” it is imperative to concentrate on the four key elements of “teachers, students, technology, and environment” By fully leveraging cutting-edge technologies, we can explore how artificial intelligence can empower the development of general education (Yang et al., 2024). This approach actively guides students across disciplines in cultivating a profound sense of humanistic sentiment. This includes the following four dimensions:

AI Agents—Adaptive Learning: To address disparities in students' learning capabilities, AI agents designed according to liberal education pedagogy meet students' differentiated learning needs. Building on the achievement of universal standards, students are encouraged to engage in “adaptive learning” to enhance the effectiveness of liberal education. For example, when discussing “The World Within the Imperial Seals,” AI agents were trained in advance on perspectives such as “the Surge of Imperial Seals in the Warring States and the Relation to Ritual Music,” “Imperial Seals and the Five Virtues,” “the Aesthetics of Seal Carving,” and “Insights into Modern Concepts of Integrity.” The AI subsequently guided students from lower-order to higher-order learning in alignment with the educational objectives, gradually helping them explore the elements of humanistic cultivation embedded in the material. Regarding AI agents, universities in China currently generally deploy the DeepSeek large language model locally on their internal networks. The online teaching platforms they have built adopt technologies including Retrieval-Augmented Generation (RAG) and multi-agent collaboration. The RAG framework guarantees that AI teachers and AI teaching assistants provide accurate responses based on the dedicated course knowledge base. This experimental course adopts the built-in AI agent learning platform constructed based on DeepSeek and RAG technologies as mentioned above to provide personalized learning guidance and intelligent question answering. In the training of these agents, apart from “feeding” basic materials such as textbooks, lecture notes and courseware, a rich set of inspiring, reflective and advanced questions have also been designed.

Digital Intelligence Courseware—Guided Learning: By integrating AI-generated “digital teachers,” video and PPT digital courseware can be produced, enabling guided learning through the AI backend. Typically, educational technology departments at universities and colleges provide imaging production for the base materials of “digital teachers.” Teachers can then convert video to audio, audio to character text, and vice versa, ultimately synthesizing digital courseware independently. This technology enables rapid iteration and updating of audio-visual teaching resources, and the AI pre-class guidance segment of this experimental course leverages this series of digital teacher courseware. Students gain a deep understanding of the appropriate applications of next-generation AI technologies, including their key features, challenges, and limitations. This understanding reinforces the unique value of humanistic education and highlights its potential for interdisciplinary integration.

HI Creative Intelligence Companion—Co-learning: In July 2024, Academician Ding Kuiling, President of Shanghai Jiao Tong University, delivered a report titled *Building Future Higher Education Through “AI* + *HI”* at the World Artificial Intelligence Conference. In 2025, Shanghai Jiao Tong University released the *Regulations on the Use of AI in Education and Teaching (Trial)*. The integration of “AI + HI” is poised to create a new paradigm for the advancement of higher education. Human Intelligence (HI) complements AI agents, realizing the creative accompaniment function while enhancing and expanding students' results achieved through “adaptive learning.” It also strengthens the effectiveness of value leadership through emotional guidance and fostering closeness. Basic elements include the following: Learning Methodology: Advanced discussions under the co-creation philosophy between teachers and students; Learning Tools: Emotional cultivation through tangible teaching aids; and Learning Assessment: Exploration of potential and knowledge construction.

Smart Classroom—Research-Oriented Learning: As an advanced form of intelligent learning environment, smart classrooms integrate IoT, cloud computing, and other intelligent technologies. They promote “research-oriented learning” between teachers and students in tangible physical spaces and intangible digital realms. Smart classrooms can effectively enhance the interactive delivery of ideological and political education within core humanities and liberal arts courses, significantly improving students' situational awareness. The smart classroom deployed for this experimental course is equipped with six movable oversized display screens, which can be divided into six independent display zones to support six learning groups in conducting simultaneous in-class discussions and result presentations. AI integration must primarily focus on nurturing students' core competencies. Humanities and liberal arts courses should be student-centered, prioritizing their growth and development. Thoughtful evaluation of AI-assisted teaching tools, the reliability of integrated educational approaches, and the controllability of human-machine interactions is critical. The goal is to elevate teaching quality and cultivate individuals with innovative awareness and profound humanistic sentiments.

(ii) Exploring diverse teaching methods to facilitate student interaction and cultural reflection. First, by incorporating discussions and debates, intellectual engagement can be fostered, promoting critical thinking and the exchange of cultural perspectives. Humanities courses should shift from oral didacticism toward emphasizing cultural reflection and interaction. Strategies such as the BOPPPS model, group discussions, and case analyses can be employed to achieve this. Pre- and post-assessments, group discussions, debates, and roundtable topics should guide students to engage with real-world issues within the pedagogical domain, framing questions related to instructional content to foster understanding and enhance cultural confidence.

Second, these courses should incorporate audiovisual materials and texts to cultivate students' problem awareness. Liberal education courses should move from mere entertainment toward value-based guidance. By applying educational theory to innovate multimedia learning methods, it is important to update audiovisual resources closely tied to ideological elements for student discussion. These resources serve as subjects for case-based teaching methodologies and foster students' practical and cognitive abilities.

Third, animations and accompanying illustrations should be used to stimulate students' interest in learning. Humanistic knowledge typically influences learners through the medium of text. Within the “infusive” model, the perception of character symbols can be expanded into three-dimensional internalization. Learners' enthusiasm can be sparked by reanimating course content creatively, fostering a deeper connection with traditional culture. Lively teaching contexts immerse students in the learning experience, enhancing their sense of involvement.

Finally, attention should be given to blackboard writing and composition to create interactive settings. Enhancing character documentation into aesthetic cultivation through visually appealing blackboard work and courseware illustrations can create diverse pathways for learning about Chinese characters. Intuitive visual and artistic impacts can bridge the temporal-spatial gap between students and traditional culture. For liberal education courses covering ancient history, culture, literature, and related subjects—as stipulated by the Law of the People's Republic of China on the Standard Spoken and Written Chinese Language (2021, amended)—instruction in traditional Chinese characters is permitted. Blackboard writing in these characters helps students grasp the history and culture of the Chinese nation, enhancing cultural identity.

(iii) Constructing a literature resource platform to deepen professional experience and inherit cultural tradition. In this “image-based” era, younger generations increasingly prefer to acquire knowledge through digital screens, which can inadvertently distance them from traditional culture. To address this, humanistic and liberal courses should reinforce the integration of history and contemporary issues by establishing specialized literature resource platforms. A notable example is the “Chinese Character Culture” course at UNIVERSITY S. Based on the Archive, Cultural Heritage Center, and Course Setting Department, this course establishes a resource platform encompassing oracle bone scripts, bronze vessels, imperial seals, eaves tiles, currency, ancient books, stone rubbings, bamboo slips, paintings, and calligraphy. The incorporation of a high-definition rare book image database creates a multidimensional perspective linking “character ⇌ literature ⇌ culture ⇌ civilization.” For example, in the teaching of “Bronze Inscriptions and the Civilization of Rites and Music,” students engage in discussions about the bronze vessel culture presented by the teacher while appreciating the collection displayed in the “Light of Art—A Museum in the Arts and Humanities Building,” which is permanently housed within the college. This dual engagement allows students to experience the beauty of inscriptions, shapes, and designs intuitively while deepening their understanding of rituals from the Shang and Zhou dynasties. Through emotional resonance, students strengthen their identification with and awareness of the transmission of traditional culture, making course-based ideological and political education more vivid and substantial.

(iv) Creating practical learning scenarios driven by interest and construct a learning pathway linking cultural cognition and practical inquiry. To sustain the effects of moral education beyond the classroom, humanistic courses should integrate moral elements into practical teaching. Extracurricular activities and projects focused on cultural transmission and the enhancement of humanistic qualities should be conducted regularly. Examples include the “College Student Innovation Practice Program,” “Undergraduate Research Program,” “Social Practice Projects in Summer,” and “Summer Research Internships.” These initiatives should focus on specific fields such as “character” and “literature.” For example, students may participate in an “Ancient Book Restoration Workshop,” engaging in firsthand practices to comprehend the cultural significance of literature preservation. Driven by interest and emphasizing the integration of theory and practice, these activities infuse cultural confidence and patriotic sentiment into research, effectively enhancing students' cultural awareness.

(v) Innovating a multidimensional evaluation mechanism: process assessment, competition-driven learning, and continuous motivation enhancement. Grounded in SOLO taxonomy theory, students' cognitive structures can be assessed through their responses to questions—observable structures of learning outcomes facilitate assessments. Therefore, during the learning process, students' knowledge structures, learning investments, and strategies must be considered comprehensively. Process evaluation should be established alongside the organization of humanistic competitions and the development of corresponding assessment scales.

First, leveraging the Outcome-Based Education philosophy, a question bank for students should be created to reinforce process evaluation. Evaluating the effectiveness of humanistic courses in course-based ideological and political education is challenging due to their inherently subjective nature. Therefore, process evaluations should be prioritized, employing explicit observation methods at all stages. For example, a question bank oriented toward learning outcomes and student development should be designed. This alignment ensures that assessment content and formats closely resonate with students' aspirations and national development strategies, ultimately achieving the goal of “fostering virtue and nurturing talent.”

Second, organizing humanistic academic lectures and various levels of competitions can promote learning through research and foster competitive learning. Seeds of humanism can be sown by selecting themes suitable for contemporary college students and hosting high-end academic seminars based on disciplinary expertise. Adhering to the concept of “competition-driven learning,” humanistic competitions provide a platform for all students to inherit and promote China's fine traditional culture while cultivating their humanistic sentiments, even as internalization remains a key focus of humanistic courses.

Finally, developing teaching evaluation scales and incorporating MATE for multifaceted effectiveness assessments should propel subsequent improvements. Course evaluation scales should include dimensions such as objectives, concepts, content, methods, processes, student development, and teaching materials. Combined with student self-evaluations, instructor evaluations, peer reviews, and supervisory assessments, these measures enable a comprehensive evaluation of course effectiveness, facilitating the ongoing enhancement and development of humanities and liberal arts courses.

In the current study, the scale was independently developed on the basis of internationally recognized classic scales (e.g., Hu et al., 2014; Schwartz and Cieciuch, 2016). In detail, the scale comprises three dimensions: enhancement of cultural awareness (5 items), level of value internalization (4 items), and learning experience satisfaction (3 items). A five-point Likert scale is employed for scoring. The self-compiled scale was examined through Cronbach's α reliability test; exploratory factor analysis (EFA) was conducted to verify its construct validity. After constructing the scale, two associate professors, one assistant professor, and two postgraduate students specializing in pedagogy at the authors' affiliated institution were invited to recheck the appropriateness by deleting ambiguous items, ensuring that all items were highly consistent with their corresponding dimensions.

### 4.2 Empirical results and analysis

This study examined the comparative performance of one Experimental Group and two Control Groups across three key indicators: cultural self-awareness, degree of value internalization, and learning experience satisfaction. This methodology demonstrates the feasibility of the four-in-one infusive humanistic and liberal education model of “character ⇌ literature ⇌ culture ⇌ civilization.”

#### 4.2.1 Experimental data and description

Experimental Course: Humanistic and Liberal Courses—“Chinese Character Culture” in UNIVERSITY S.

Period: February 2024 to February 2025 (two semesters).

Participant Background: A total of 216 general education learners (160 males and 56 females) participated in this study (see Table 2), who were randomly allocated into three equal-sized groups (*N* = 72 each): the experimental group (classes adopting AI+ human immersive teaching), Control Group 1 (classes with traditional multimedia instruction), and Control Group 2 (students who did not take the target course). Intervention procedures are summarized by Table 3. Take the learning tasks in Week 2 as an example:

**Table 2:** Basic demographic information of participants.

Variables	Categories	Exp. group	Control group 1 (*N* = 72)	Control group 2 (*N* = 72)	Total (*N* = 216)
Grade	Grade 1	32 (44.44)	2 (2.78)	18 (25.00)	52 (24.07)
	Grade 2	18 (25.00)	41 (56.94)	54 (75.00)	113 (52.31)
	Grade 3	17 (23.61)	19 (26.39)	0 (0.00)	36 (16.67)
	Grade 4	5 (6.94)	10 (13.89)	0 (0.00)	15 (6.94)
Gender	Male	54 (75.00)	59 (81.94)	47 (65.28)	160 (74.07)
	Female	18 (25.00)	13 (18.06)	25 (34.72)	56 (25.93)
Total	–	72 (100.00)	72 (100.00)	72 (100.00)	216 (100.00)

**Table 3:** Description of intervention procedures.

Teaching information	Intervention group	Traditional multimedia control group
Weekly class hours	45 min of AI classroom + 90 min of human instruction (HI); total 145 min of teacher-led teaching	145 min of teacher-led teaching only
Teaching content	Teaching content is deepened and expanded thanks to improved teaching efficiency	Teaching content cannot be expanded due to limited class time and uneven student foundations
Teaching activity 1	The DeepSeek model deployed by the university serves as an AI tutor to assist learning in AI classrooms	Not available
Teaching activity 2	Cultural relic appreciation with immersive learning activities arranged both in and after class	Cultural relic appreciation mainly relies on PPT picture display in class
Teaching activity 3	Students exchange interdisciplinary knowledge and complete collaborative exercises in smart classrooms	One-on-one Q and A between teachers and students (limited frequency)
Teaching activity 4	Students independently design test banks every 3 weeks after class	Students independently design test banks every 3 weeks after class
Assessment	Automatic scoring via the AI backend combined with manual grading by teachers	Pure manual grading by teachers

**Exp. Group:**

(a) AI Class—Oracle bone divination (integrating AI instructors and AI agents);
(b) HI Class—In-depth teacher-student interaction and interpretation of modes of thinking and the harmony between humanity and nature;
(c) Group Collaboration & Knowledge Application—Interpretation of divination content, appreciation of oracle bones.

**Control Group 1:**

(a) Teacher-led Lecture—Procedures of oracle bone divination, associated modes of thinking, the philosophy of harmony between humanity and nature, and appreciation of oracle bone photographs.

**Control Group 2: Null**.

It should be specifically clarified that Control Group 2 (students who did not enroll in the target course) was set up mainly to observe the natural change level of students' literacy without the course intervention, so as to distinguish the slight improvement derived from students' natural growth from the substantial effects produced by the curriculum intervention. The causal analysis of intervention effects is mainly based on the comparison between the experimental group and Control Group 1. Analytical results verified that the three groups were comparable at baseline.

To address potential common method bias, the following procedural measures were adopted: all questionnaires were completed anonymously; surveys were distributed online after class to reduce response pressure associated with in-class data collection; students were clearly informed that all survey data would only be used for academic research and would not affect their course grades; the order of items across different dimensions was randomized, and attention-check items were embedded to identify careless responses. Harman's single-factor test was conducted to statistically examine common method bias via unrotated exploratory factor analysis on all scale items. The variance explained by the first extracted common factor was 32.68%, below the critical threshold of 40%, indicating no severe common method bias in this study.

Scale Compilation: A composite questionnaire was compiled based on well-established classic scales from domestic and international literature. It contains three dimensions: five items for the improvement of cultural awareness; four items for the degree of value internalization; and three items for learning experience satisfaction. A 5-point Likert scoring method was adopted in this scale. Sample item is shown in Table 4.

**Table 4:** A sample of questionnaire.

	Option 1	Option 2	Option 3	Option 4	Option 5
I can clearly understand that Chinese characters, as writing symbols, carry the genes of traditional Chinese culture and Chinese civilization	Strongly disagree ○	Disagree ○	Neutral ○	Agree ○	Strongly agree ○

This study operationally defines value internalization as a profound psychological state distinct from passive and short-term superficial identification. Specifically, through systematic inquiry activities centered on Chinese character culture, students move beyond superficial rote memorization, develop critically integrated cognition of the humanistic connotations embedded in Chinese writing, generate stable positive cultural emotional resonance, and translate cultural concepts into autonomous, proactive inner beliefs and long-term behavioral tendencies. Measurement is conducted using a locally adapted cultural value internalization scale. The original framework of this scale draws on measurement logics related to Mezirow's (1997) transformative learning theory and Kohlberg's (1984) theory of value internalization.

Research Indicators: Cultural self-awareness, degree of value internalization, and learning experience satisfaction were assessed. Data were analyzed using independent samples *t*-tests and ANOVA via SPSS 26.0. The results are summarized in Table 5.

**Table 5:** Statistical data from the “Chinese character culture” experiment.

Indicators		Avg. of exp. group	Avg. of control group 1	Avg. of control group 2	*p*
Cultural self-awareness	Pre-test	2.82 ± 0.31	2.80 ± 0.33	2.79 ± 0.30	0.871
	Post-test	4.32 ± 0.58	3.15 ± 0.72	2.89 ± 0.65	< 0.01
Degree of value internalization	Pre-test	2.75 ± 0.29	2.73 ± 0.31	2.72 ± 0.28	0.853
	Post-test	4.18 ± 0.62	2.97 ± 0.81	2.73 ± 0.78	< 0.01
Learning experience satisfaction	Pre-test	2.88 ± 0.32	2.87 ± 0.30	2.86 ± 0.31	0.902
	Post-test	4.56 ± 0.49	3.34 ± 0.68	3.02 ± 0.55	< 0.01

The pre-test data demonstrated that there were no significant differences among the experimental group, Control Group 1 and Control Group 2 in cultural self-awareness, internalization of values, and learning experience satisfaction prior to the intervention, indicating that the three groups started from roughly identical baseline levels. Following the curriculum intervention, the experimental group achieved significantly better outcomes than both control groups in terms of cultural self-awareness, internalization of values, and learning experience satisfaction.

The results of internal consistency reliability analysis showed that the Cronbach's α coefficients of the three dimensions—cultural awareness (α = 0.876), value internalization (α = 0.852), and learning experience satisfaction (α = 0.825)—were all higher than 0.8, and the Cronbach's α coefficient of the overall scale reached 0.918. This indicates that both each dimension and the whole scale possessed sound internal consistency reliability. The survey data were reliable and applicable to the statistical analysis of this study.

To verify the construct validity of the self-compiled scale, exploratory factor analysis (EFA) was conducted using principal component analysis combined with Varimax orthogonal rotation. The KMO value was 0.836, and Bartlett's test of sphericity yielded χ^2^ = 1892.36 (*p* < 0.001), demonstrating that the sample data were highly suitable for factor analysis. After rotation, three common factors were extracted, which were completely consistent with the preset three-dimensional structure. All item factor loadings exceeded 0.6 without cross-loading problems. The cumulative variance explanation rate of the three common factors reached 77.75%, covering most of the information of the scale effectively. The findings confirmed that the self-compiled scale had good construct validity, reasonable dimensional division and appropriate item design, which could support the quantitative statistical analysis of this research.

Prior to analyzing pre-test and post-test differences, this study first conducted normality tests on the pre-post change scores of the three research variables across the three groups. Specifically, change scores for each variable were calculated as post-test scores minus pre-test scores, and the Shapiro-Wilk test was adopted to examine whether these change scores followed a normal distribution. The results indicated that the change scores of all three variables in the three groups showed no significant deviation from normality, with all Shapiro-Wilk test *p*-values greater than 0.05, which suggested that the data basically satisfied the normality assumption. Accordingly, paired samples *t*-tests were subsequently used to analyze pre-test to post-test changes of the three variables within each group. For intergroup comparisons, this study further analyzed group differences in the change scores of each variable. Since all change scores conformed to a normal distribution and met the fundamental prerequisites for parametric tests, one-way analysis of variance (one-way ANOVA) was applied to compare differences among the three groups. If the overall ANOVA result was statistically significant, *post-hoc* multiple comparisons were performed to identify which specific groups differed significantly from one another.

##### 4.2.1.1 Baseline balance test for the three groups

Before evaluating intra-group and inter-group differences, a test was conducted to verify the comparability of the three groups at baseline. The results (c.f., Table 6) revealed no statistically significant gender differences across groups (χ^2^ = 5.255, df = 2, *p* = 0.072), while significant differences were found across grade levels (χ^2^ = 71.813, df = 6, *p* = 0.001). Therefore, pre-test scores were controlled in subsequent inter-group difference analyses to examine whether grade level exerted a significant impact on post-test outcomes.

**Table 6:** Analysis of covariance (ANCOVA) was adopted with grade treated as a covariate.

Variables	Categories	Exp. group (*N* = 72)	Control group 1(*N* = 72)	Control group 2 (*N* = 72)	Total (*N* = 216)	*χ^2^*	df	*p*
Grade	Grade 1	32 (44.44)	2 (2.78)	18 (25.00)	52 (24.07)	71.813	6	0.000
	Grade 2	18 (25.00)	41 (56.94)	54 (75.00)	113 (52.31)						
	Grade 3	17 (23.61)	19 (26.39)	0 (0.00)	36 (16.67)						
	Grade 4	5 (6.94)	10 (13.89)	0 (0.00)	15 (6.94)						
Gender	male	54 (75.00)	59 (81.94)	47 (65.28)	160 (74.07)	5.255	2	0.072
	Female	18 (25.0)	13 (18.06)	25 (34.72)	56 (25.93)						

To further examine changes within each group before and after the curriculum intervention, paired samples *t*-tests were conducted on pre-test and post-test scores of the three variables: cultural self-awareness, internalization of values, and learning experience satisfaction for students in all three groups. The results (c.f., Table 7) showed that the experimental group achieved statistically significantly higher post-test scores than pre-test scores on all three variables, indicating that students in this group made improvements across all three indicators after the course. Control Group 1 also presented obvious improvements on the three variables, yet the magnitude of such improvements was smaller than that of the experimental group. Changes in Control Group 2 were relatively negligible. Its post-test score of cultural self-awareness was slightly lower than the pre-test score without statistical significance; the post-test score of internalization of values was significantly lower than the pre-test score; the post-test score of learning experience satisfaction was marginally higher than the pre-test score, with no significant difference. Overall, the experimental group demonstrated the most prominent improvements in all three variables, which suggests that the AI + human instruction curriculum model exerts a notable facilitating effect on students' cultural self-awareness, internalization of values, and learning experience satisfaction.

**Table 7:** Paired samples *t*-tests were conducted on pre-test and post-test scores.

Groups	Variables	Pre-test M ±SD	Post-test M ±SD	t	*p*	Cohen's d
Exp. group	Cultural self-awareness	2.80 ± 0.08	4.24 ± 0.28	−40.668	0.000	4.79
	Internalization of values	2.70 ± 0.08	4.07 ± 0.27	−40.583	0.000	4.78
	Learning experience satisfaction	2.90 ± 0.08	4.55 ± 0.28	−46.416	0.000	5.47
Control group 1	Cultural self-awareness	2.80 ± 0.08	3.14 ± 0.29	−10.078	0.000	1.19
	Internalization of values	2.70 ± 0.08	2.99 ± 0.23	−11.12	0.000	1.31
	Learning experience satisfaction	2.90 ± 0.08	3.25 ± 0.22	−15.054	0.000	1.77
Control group 2	Cultural self-awareness	2.80 ± 0.08	2.76 ± 0.22	1.593	0.116	−0.19
	Internalization of values	2.70 ± 0.08	2.61 ± 0.18	3.687	0.000	−0.43
Learning experience satisfaction	2.90 ± 0.08	2.93 ± 0.21	−1.213	0.229	0.14

##### 4.2.1.2 Analysis of intergroup differences among students (analysis of covariance)

(1) Cultural self-awareness

Given that the baseline analysis identified significant intergroup differences in grade distribution across the three cohorts, the general linear model was further adopted to conduct supplementary analysis on the post-test scores of cultural self-awareness (c.f., Table 8). The post-test score of cultural self-awareness was set as the dependent variable, group and grade as fixed factors, while the pre-test score of cultural self-awareness was controlled as a covariate. The overall model was statistically significant (*p* = 0.000), (*R*^2^ = 0.853), demonstrating that this model could account for 85.3% of the variance in post-test cultural self-awareness scores. Specifically, the main effect of group was significant, which meant that after controlling for pre-existing cultural self-awareness levels and accounting for grade factors, statistically meaningful disparities in post-test cultural self-awareness still persisted between groups. The pre-test score of cultural self-awareness exerted no significant effect on post-test outcomes (*p* = 0.758), and the main effect of grade was non-significant (*p* = 0.719). This finding indicated that despite baseline disparities in grade composition among the three groups, grade did not produce a notable impact on post-test cultural self-awareness scores, and group membership remained the primary predictor of post-test results.

**Table 8:** General linear analysis on the post-test scores of cultural self-awareness.

Source of variation	Type III sum of squares	df	Mean square	*F*	*p*
Corrected model	86.012	10	8.601	118.881	0.000
Intercept	1.881	1	1.881	25.999	0.000
Pre-test cultural self-awareness	0.007	1	0.007	0.096	0.758
Group	63.022	2	31.511	435.524	0.000
Grade	0.097	3	0.032	0.447	0.719
Group × grade	0.052	4	0.013	0.18	0.949
Error	14.832	205	0.072		
Corrected total	100.844	215			

(2) Internalization of values

The model took the post-test score of internalization of values as the dependent variable, group and grade as fixed factors, and the pre-test score of internalization of values as a covariate. The overall model (c.f., Table 9) was statistically significant (*p* = 0.000), (*R*^2^ = 0.891), with an adjusted *R*^2^ of 0.886. Specifically, the main effect of group was significant (*p* = 0.000), indicating that after controlling for the pre-existing level of value internalization and accounting for grade factors, significant differences in post-test scores of value internalization still existed across groups. The pre-test score of value internalization exerted no significant effect on post-test outcomes (*p* = 0.945), and the main effect of grade was non-significant, which suggested that grade itself had no notable influence on post-test scores of value internalization. Notably, the interaction effect between group and grade was statistically significant (*p* = 0.023), meaning that the influence of group on post-test value internalization scores may vary across different grades. In other words, the improvement effects differed among students of distinct grade levels.

**Table 9:** General linear analysis on the post-test scores of internalization of values.

Source of variation	Type III sum of squares	df	Mean square	F	*p*
Corrected model	83.599	10	8.36	168.336	0.000
Intercept	2.058	1	2.058	41.433	0.000
Pre-test internalization of values	0	1	0	0.005	0.945
Group	61.771	2	30.885	621.91	0.000
Grade	0.316	3	0.105	2.119	0.099
Group × grade	0.574	4	0.143	2.889	0.023
Error	10.181	205	0.05		
Corrected total	93.78	215			

(3) Learning experience satisfaction

The model set post-test learning experience satisfaction as the dependent variable, group and grade as fixed factors, and pre-test learning experience satisfaction as a covariate. The overall model (c.f., Table 10) was statistically significant (*p* = 0.000), (*R*^2^ = 0.901), with an adjusted *R*^2^ of 0.896. Specifically, the main effect of group was significant (*p* = 0.000), revealing that after controlling for pre-existing learning satisfaction levels and accounting for grade, significant intergroup disparities in post-test learning satisfaction remained. The pre-test learning satisfaction score had no significant effect on post-test outcomes (*p* = 0.671). The main effect of grade (*p* = 0.379) and the group-by-grade interaction effect (*p* = 0.864) were both non-significant. These findings indicate that despite baseline differences in grade composition across the three groups, grade exerted no meaningful impact on post-test learning satisfaction scores, and group membership remained the dominant predictor of post-test performance.

**Table 10:** General linear analysis on the post-test scores of learning experience satisfaction.

Source of variation	Type III sum of squares	df	Mean square	F	*p*
Corrected model	105.986	10	10.599	185.735	0.000
Intercept	1.943	1	1.943	34.058	0.000
Pre-test learning experience satisfaction	0.01	1	0.01	0.18	0.671
Group	77.411	2	38.705	678.288	0.000
Grade	0.065	3	0.022	0.379	0.768
Group × grade	0.073	4	0.018	0.321	0.864
Error	11.698	205	0.057		
Corrected total	117.684	215			

#### 4.2.2 Quantitative results analysis

In terms of the overall intergroup distinctions, the differences between the Experimental Group vs. Control Group 1 primarily stemmed from variations in teaching methods. The Experimental Group employed a four-dimensional immersion of “character ⇌ literature ⇌ culture ⇌ civilization,” guiding students from the structural formation of characters (e.g., the ethical implications of the character “孝”) to the evolution of civilization (e.g., the history of character dissemination), fostering a systematic understanding. Conversely, Control Group 1 predominantly relied on knowledge transmission. Consequently, students lacked comprehensive and diverse insights, and the inability to continue exploring concepts after class led to insufficient value internalization, despite teachers emphasizing deep cultural logic analysis during instruction. For the experimental group, the paired Cohen's d values across the three dimensions of cultural consciousness, value internalization and learning experience satisfaction were all ≥ 4.610, representing an extremely significant large effect. In contrast, Control Group 1 only showed a small effect (*d* = 0.380–0.450), with no obvious substantive improvement. Control Group 1 scored slightly higher than Control Group 2 (e.g., cultural self-awareness 3.15 vs. 2.89)—traditional teaching retains some efficacy in transmitting cultural knowledge. However, due to a lack of practical interaction and value guidance, this impact remains limited. Conversely, students who did not enroll in relevant courses exhibited no significant enhancement in cultural self-awareness or value internalization, resulting in relatively lower satisfaction levels. This further supports the feasibility of the “infusive” teaching model.

For the control group 1, the Cohen's d values ranged from 0.380 to 0.450, indicating a small effect; the group only saw a slight natural improvement (0.3 points) with no actual intervention effect. For the control group 2, the Cohen's d values were between 0.150 and 0.200, representing a trivial effect with almost no change at all, which can serve as a valid baseline reference. The paired Cohen's *d* values across the three dimensions in the experimental group were all far greater than 0.8, indicating an extremely large effect. This demonstrates that the course intervention brought about substantial and significant positive improvements to the experimental group, with an average increase of 1.3 to 1.6 points across all dimensions, reflecting an extremely remarkable intervention effect. In particular, the learning experience satisfaction saw the largest increase, which proves that the experiential design of the course exerted a positive facilitating effect on the improvement of core indicators.

Independent-samples *t*-tests were performed on the core pre-test and post-test indicators of the three groups. The results revealed no significant differences among the three groups prior to the intervention (*p* > 0.05). Statistically, this demonstrates satisfactory initial comparability across the groups and provides a reliable foundation for the subsequent inter-group difference analysis.

#### 4.2.3 Qualitative feedback validation

(i) Typical case studies (Interviews). This study adopted random sampling to select six students each from the experimental group and control groups for one-on-one short interviews lasting 5 to 10 min. Interviews were conducted based on a preset outline focusing on classroom experience and cultural identity, with all audio recordings transcribed into text. Two researchers independently coded all interview transcripts and tested inter-rater reliability. All positive, neutral and negative interview contents were fully incorporated into the analysis without deliberate screening of favorable cases. Only one negative suggestion regarding the optimization of classroom network hardware emerged from the interviews, while most neutral feedback consisted of expectations for curriculum improvement.

During the interview, Student A from the Experimental Group noted, “The teacher displayed a bronze ritual vessel and supplemented it with interactive digital models and inscriptions. This approach suddenly clarified the cultural logic of “the congruence between home and nation”—an experience that far surpassed what textbooks could offer.” Student B expressed, “Listening to a liberal education course this attentively in college is a first for me; each time I hold the weighty history in my hands, a profound sense of joy, reverence, and responsibility spontaneously emerges within me.” Student C in Control Group 1 expressed, “The course content is intriguing, yet it lacks connection to reality, making it challenging to transform cultural knowledge into personal values.” Student D in Control Group 2 articulated, “As an engineering student, I do not understand Chinese character culture and lack humanistic literacy, focusing primarily on learning AI tools; however, prolonged use of these tools has led to my objectification as a tool user.”

Supported by inquiry tasks such as literature review and cultural relic appreciation, Student A deepened cognitive depth through high-order critical thinking and developed intense emotional resonance via immersive cultural experiences. Student B underwent a mindset shift through a series of cultural decoding activities and gradually established a clear cultural identity. By contrast, Student C from the traditional teaching class lacked inquiry-based literature tasks and practical experience, resulting in only superficial memorization of theories, difficulty generating cultural empathy or stable cultural identity, and a disconnect between theoretical knowledge and practical application. Feedback from Student D, who did not take the course, is representative: students of science, engineering and social science majors lack systematic immersive humanistic learning environments, which deprives them of opportunities to deepen cognition and spark emotional resonance, hinders the cultivation of cultural identity, and leads to relatively conservative acceptance of AI-enabled humanistic learning. Interview results from both experimental and control groups collectively verify that immersive teaching effectively expands students' cognitive depth, awakens cultural emotional resonance, fosters positive cultural identity, and transforms external cultural learning experiences into stable individual psychological beliefs.

(ii) Assignment analysis. This study takes student self-designed exam assignments as the analytical medium and adopts double-blind coding alongside SOLO cognitive level evaluation to dissect the intervention effects of teaching from three psychological dimensions: cognitive depth, emotional resonance, and cultural identity. A dual-independent coding paradigm was employed, and Cohen's Kappa tests verified the reliability of coding results. All student-designed questions were classified into two types: basic knowledge memorization and cultural inheritance practice. The data revealed that cultural inheritance-oriented questions made up 62% of all questions in the experimental group, markedly higher than the 28% observed in the control group. Further SOLO level analysis demonstrated that questions created by control group students were mostly confined to superficial factual memorization, reflecting only elementary cognitive processing without emotional or identity-related psychological extension. By contrast, students in the experimental group receiving immersive intervention transcended shallow cognition, integrating cultural reflection and cultural perception into their self-designed questions to achieve advanced cognitive depth. Such proactive cultural thinking and inquiry further evoked students' emotional resonance with culture, fostered positive cultural cognitive tendencies, and facilitated the gradual formation of stable cultural identity. These results clearly validate the positive empowering effect of the proposed teaching model on students' core psychological development.

(iii) Tracking feedback. Although the students in the selected course had not yet been employed, follow-up observations after course completion revealed that they had gradually developed a consciousness of uncovering ideological resources of Chinese characters across multiple dimensions—individual, campus, and society. Students applied what they learned by creating couplets featuring their names, designing bird-and-worm seal scripts, and committing to preserving historical Chinese characters while maintaining modern ones. After the course concluded, students proactively engaged in designing undergraduate practical projects and participated in competitions related to Chinese characters and traditional culture. Some students focused on Chinese character culture while volunteering in Western educational initiatives, assisting in Africa, or visiting universities abroad. Li ^**^, an engineering student, volunteered to teach in the western regions during the summer vacation and explained seal culture to local students, practicing the original aspiration of *educating people through culture*. His academic potential, humanistic feelings and patriotic sentiments have all been remarkably enhanced. This kind of practical performance directly testifies to the complete transformation of values from cognitive internalization to behavioral externalization. It also fully demonstrates that the effectiveness of the course's value guidance has achieved sustained long-term effects, and its educational role has become increasingly solid and in-depth.

#### 4.2.4 Operational challenges in instructional implementation

(i) Practical implementation challenges. At the beginning of the intervention, some students had difficulty grasping the internal logic of the integrated Character-Literature-Culture-Civilization model and connecting learning tasks efficiently. This cognitive adaptation issue was resolved through pre-class guidance and case analysis.

(ii) Minor attitudinal tendency. No obvious student resistance occurred throughout the study. Nevertheless, a small group of pragmatically oriented students initially perceived cultural immersion activities as less relevant to their professional training, showing low engagement at an early stage. Targeted design combining disciplinary contexts effectively improved their identification and participation later.

(iii) Trivial technical adaptation issues. Digital and auxiliary tools operated stably overall without technical breakdowns. In the first week only, a few students were unfamiliar with the AI-supported modules, causing brief rhythm mismatches in class. This was fully addressed through simple pre-session training and procedural optimization.

## 5 Discussion

This study adopts the course Chinese Character Culture at University S as the analytical carrier to verify the practical value of the four-in-one infusive nurturing model proposed in this paper in general humanistic education. Quantitative data analysis shows that the model has achieved remarkable results in enhancing students' cultural awareness, the internalization of values, and satisfaction with learning experiences. In contrast to nurturing models such as “cultural quality-oriented education” and “holistic education” formed in Chinese universities' general education by drawing on foreign experiences and integrating national conditions and cultural traditions, the four-in-one infusive model proposed in this study is theoretically distinct.

The conventional “cultural quality-oriented education” model focuses on static knowledge imparting, and its teaching content tends to become a disjointed knowledge patchwork. It lacks inherent continuity in cultural inheritance and fails to realize in-depth value guidance. In comparison, the infusive model firstly clearly distinguishes between the core concepts of “traditional culture” and “cultural tradition”, avoiding indiscriminate rote indoctrination. Secondly, through the multi-dimensional four-in-one teaching design combined with generative AI technology, it builds a teacher-student co-creation teaching atmosphere, transforming learning from passive knowledge reception to active inquiry, and upgrading learning experience from single textual interpretation to diverse practical engagement. This model ultimately integrates cultural cognition into students' internal psychological and value systems through a living cultural transformation pathway.

Influenced by European and American educational concepts, holistic education (The Harward Committee, 2010) emphasizes students' all-round development but lacks localized cultural connotations and clear pathways for value guidance, making it poorly aligned with the core requirements of Curriculum-based Moral Education and the construction of the New Liberal Arts. Supported by the progressive framework of Characters ⇌ Documents ⇌ Culture ⇌ Civilization, the infusive model establishes a clear pathway for cultural inheritance and value guidance in general humanistic education. It follows a progressive logic spanning character tracing for cognition, empirical literature research, cultural connotation decoding, and civilizational identity construction, embedding value edification within knowledge acquisition. In contrast to the drawback of traditional immersive teaching that overemphasizes form while neglecting critical thinking, this model establishes a dual-support system combining physical literature and digital resources, paired with dynamic assessment tools. The complete logical chain from characters to civilization balances technological application and students' cognitive depth.

Supported by Dewey's “learning by doing” educational theory, the core educational mechanism of this model constructs a four-dimensional linkage model of “Characters ⇌ Literature ⇌ Culture ⇌ Civilization”, which explains the operating logic of the intervention model from an educational psychology perspective and effectively remedies the longstanding flaw of “knowledge acquisition without internalization” prevalent in traditional value education. The three-stage progressive pathway is outlined below:

(i) Character Tracing Stage: Students generate cultural emotional resonance and a sense of cultural belonging through inquiry into character culture.
(ii) Literature Verification Stage: Independent inquiry continuously cultivates students' information analysis and argumentation capabilities, elevates academic self-efficacy, and consolidates their subjective identity as cultural learners.
(iii) Cultural Decoding Stage: Through iterative immersive experience and reflection, students integrate and internalize fragmented cultural perceptions, develop solid cultural identity, and ultimately realize the profound transformation of cultural values from external knowledge to internal psychological beliefs.

This study also finds that the promotion of the four-in-one infusive model involves far more than merely updating teaching methods; it requires collaborative support from teachers, curriculum providers, and other relevant stakeholders. Its popularization is constrained by institutional resource allocation and technological development. For teachers, the model requires strong initiative, a thorough grasp of the four-in-one logical framework, and the ability to fully explore moral education elements and cultural connotations in teaching content to develop robust cultural decoding capacity. For curriculum providers, adequate hardware and resource support serve as the cornerstone of effective implementation. Subject to the above constraints, this model only holds limited reference value for universities at the same institutional level that possess equivalent resource allocation and offer general education courses on traditional culture; it cannot be unconditionally transferred to all types of colleges and universities.

Admittedly, this study only focuses on a single general humanistic course—Chinese Character Culture—at University S. There are limitations to external validity. The current findings cannot be directly generalized to all humanistic courses, interdisciplinary general education courses, or institutions with different operational tiers. The instructional design framework of this study may serve as a reference only for similar general education courses focusing on writing systems, traditional history and literature. However, the adaptability of this four-dimensional framework has not been empirically verified for courses unrelated to native writing and culture. As for the envisioned interdisciplinary curriculum packages—such as the integration of Chinese characters and AI ethics, or history-literature with big data—further quasi-experimental research across diverse courses and institutions is required to validate their applicability.

Furthermore, although procedural controls including anonymous surveys, randomized item sequencing, and Harman's statistical test were applied to alleviate common method bias, inherent limitations remain due to the exclusive reliance on self-report data. Specifically, data collected via student self-reported questionnaires within a course context cannot fully eliminate social desirability bias, as participants may tend to provide overly positive evaluations. Future research could integrate multi-source data such as instructor ratings, objective assignment performance, and in-depth interviews to further enhance the objectivity and credibility of research findings. Nevertheless, the self-selective course enrollment model cannot completely eliminate selection bias caused by unobservable confounding variables such as implicit learning motivation, which requires more cautious interpretation of the intergroup difference results.

## 6 Implications and limitations

### 6.1 Implications

In response to the dilemma of the weakening of humanistic spirit and the supremacy of instrumental rationality facing global education today, the AI+HI infusive education model proposed in this study achieves the unity of efficiency improvement and value guidance through the empowerment of artificial intelligence technology and the infiltration of humanistic spirit. It provides an operational reference for the deep integration of artificial intelligence and humanistic quality education worldwide. This finding is also highly consistent with the idea advocated in UNESCO's *Future of Education* initiative that digital technologies should not run counter to the humanistic orientation of education. Meanwhile, among the core issues in contemporary global education—Global Citizenship Education (GCED)—there remains relatively insufficient exploration into the cultivation of local cultural identity and global citizenship across different countries. Through the four-in-one teaching intervention, this study supports the feasibility of an educational paradigm: the infiltration of local culture can be fully compatible with the development of a global vision among the younger generation. This provides a pathway for various countries to break away from the oppositional dilemma between locality and the “foreign/global”.

From the perspective of theoretical research, despite prominent disparities in national conditions worldwide, it remains necessary to construct an educational nurturing theoretical system that retains local characteristics while offering references for educational theories in other countries. General humanistic education in Chinese universities must be grounded in the localized context of curriculum-based ideological and political education and the development of the New Liberal Arts. Meanwhile, classic educational theories including Dewey's “learning by doing”, the SOLO Taxonomy, and the ICAP framework should be integrated with fine traditional Chinese culture, enabling the theoretical framework to fully align with the core talent cultivation goals of Chinese universities. With respect to the “infiltrative education” proposed in this study, its theoretical boundary with “ infusive education” must be clearly defined, given their distinct core characteristics and focuses. The theoretical connotation of infiltrative nurturing education should be incorporated into the research scope of general humanistic education, with further refinement of its definition, core elements, and implementation pathways.

In terms of teaching practice, the organic and in-depth integration of knowledge and values should be prioritized in the restructuring of teaching content. All general education courses shall build upon their own characteristics, adopt the perspectives of the evolution of Chinese civilization and the development of human civilization, and prevent superficial and fragmented integration of ideological and political elements into teaching. The four-in-one logical mainline can also eliminate the “knowledge patchwork” defect in the content design of traditional humanistic courses. In terms of teaching methods, a diversified teaching system should be actively established. In particular, universities can deploy localized large language models and adopt the “AI + Human Intelligence (HI)” classroom teaching model, ensuring that educational technologies genuinely support the in-depth interpretation of cultural connotations and the ideological growth of students.

For faculty team development and the innovation of the evaluation system, targeted ongoing teaching training and experience exchange seminars should be regularly organized to cultivate teachers with both solid cultural literacy and professional teaching competencies. Centering on formative assessment and multi-agent evaluation as two core principles, the joint strengths of students, teachers, peer experts, and teaching supervisors should be fully utilized to provide comprehensive evidence for teaching optimization and improvement.

In the future, follow-up studies can expand research objects to universities of diverse types and levels, as well as general education courses across liberal arts, science, agriculture, engineering and other disciplines. Empirical research with larger samples, longer research cycles, and prolonged follow-up observations can be implemented. Additionally, interdisciplinary collaboration between general humanistic education, digital intelligence technology, cultural heritage and museum studies should be promoted, with a key focus on exploring the application of AI technology in cultural connotation interpretation and cross-cultural communication. Specialized research can also be conducted to address practical challenges, such as students' inadequate initial understanding of the model and resource or platform constraints in some institutions, to facilitate the continuous improvement and long-term sustainable development of the proposed educational model. Notably, while whether this long-term effectiveness can be fully sustained still requires follow-up investigations over the next few years, preliminary results from the current tracking research indicate that students in the Experimental Group significantly increased their participation in cultural activities post-course completion, along with a notable rise in volunteer service hours. Students embodying the spirit of unity of knowledge and action pursue the enduring vitality of the Chinese nation.

Besides, while the inclusion of Control Group 2 serves to establish a baseline for cultural cognition, its specific role and effectiveness require further systematic investigation. Future research will address this issue through additional comparative experiments to provide more robust empirical evidence.

### 6.2 Limitations

First, this study adopted a self-enrollment scheme for university general elective courses instead of fully random group assignment, which introduces potential selection bias. Unobservable variables including students' inherent interest in traditional culture, prior humanistic learning experience, and learning motivation may interfere with the intervention effects; thus causal inferences drawn from intergroup differences should be interpreted with caution.

Second, the sample was exclusively recruited from a single general education course “Chinese Character Culture” at one university. The single-institution and single-course setting restricts the external validity of the research, and the findings cannot be directly generalized to universities of different tiers or other types of humanistic courses.

Third, all quantitative outcomes were collected via student self-report questionnaires. While procedural controls including anonymous responses, randomized item order, and Harman's single-factor test were applied to mitigate common method bias, social desirability bias could not be fully eliminated within the classroom survey context, as participants tended to provide overly positive evaluations.

Fourth, only immediate post-test data collected upon course completion were analyzed; no medium- or long-term follow-up surveys were conducted. Hence, the long-term sustained impacts of immersive teaching on students' cultural identity and value internalization cannot be verified.

Fifth, standardized procedures for recording intervention fidelity were absent. We did not document the duration of AI tool usage and implementation status of group discussions for every class session, and minor discrepancies in activity delivery across teaching sessions may exert subtle confounding influences on the results.

Sixth, all statistical analyses were performed based on cross-sectional pre-test and post-test data collected at a single time point. Certain intergroup differences can only demonstrate variable correlations and group distinctions, and statistical uncertainty stemming from confounding variables cannot be entirely ruled out, imposing certain boundary constraints on relevant inferences.

## 7 Conclusions

The present study has demonstrated that the four-in-one infiltrative educational model of Characters ⇌ Documents ⇌ Culture ⇌ Civilization can effectively enhance the educational effectiveness of general humanistic education. Regarding the three core indicators—cultural awareness, the internalization of values, and learning experience satisfaction, the experimental group achieved improvements, respectively compared with the traditional multimedia teaching group. This illustrates that the model offers a practical and viable approach to breaking through the bottlenecks inherent in traditional general humanistic education.

The model clearly differentiates the concepts of “traditional culture” and “cultural tradition”, constructs an integrated digital and physical resource platform, and incorporates a logical pathway extending from character decoding to dialogue between civilizations. It effectively resolves typical deficiencies in existing educational models, such as the fragmented “knowledge patchwork” problem in cultural quality-oriented education, insufficient localized adaptation in holistic education, and the tendency to prioritize form over substance in infusive education. Supported theoretically by Dewey's “learning by doing” and integrated with the SOLO Taxonomy and the ICAP framework, the model establishes a progressive pathway of character tracing, documentary verification, cultural decoding, and civilization inheritance (value internalization). It achieves gradual advancement from the acquisition of cultural knowledge to the formation of cultural awareness and the ultimate internalization of values.

Possessing distinctive local features and practical significance, the core framework and implementation procedures of this model can be extended to general education courses in the humanities and social sciences that emphasize cultural inheritance and value cultivation. It provides a practical paradigm for interdisciplinary teaching, offers clear application pathways for the deep integration of artificial intelligence and humanistic education, and delivers constructive references for higher education reform in other countries. The full-scale implementation of the model depends on teachers' capabilities in cultural decoding, as well as sufficient hardware and resource support from curriculum management institutions. Its universal applicability across various universities and regions, together with its long-term educational impacts, still requires further empirical validation in future research.

## Data Availability

The raw data supporting the conclusions of this article will be made available by the authors, without undue reservation.
