# Supplementary material for: Innovative practices in humanistic and liberal education: a four-in-one “infusive” educational model regarding character, literature, culture, and civilization
Source: Front Psychol. 2026 Aug 6;17:1905343. doi: 10.3389/fpsyg.2026.1905343 (PMC13491033; doi:10.3389/fpsyg.2026.1905343)
Supplement: Supplementary file 1 [file Data_Sheet_1.docx]

**Supplementary materials for submission**

**Innovative practices in humanistic and liberal education: A four-in-one “infusive” educational model regarding Character, Literature, Culture, and Civilization**

**1. Procedures for Scale Adaptation in This Study**

(1). Literature Review: Mature scales related to humanistic education, cultural self-awareness, and curriculum ideological and political education were systematically retrieved, and an initial pool of 25 candidate items was collected.
(2). Item Screening: Based on Chinese and English scale items and combined with the immersive curriculum scenario of this study, items inconsistent with domestic teaching contexts, obscure in expression, or repetitive were deleted, leaving 18 preliminary items.
(3). Expert Review: Two associate professors of pedagogy and one assistant professor of education reviewed the 18 initial items in terms of content suitability, expression clarity, and dimensional affiliation. Four items with ambiguous wording or irrelevant to the research theme were removed, leaving 14 items.
(4). Small-Scale Pilot Test: A pilot survey was conducted among 35 students from the same university. Items with low reliability were eliminated according to corrected item-total correlation (CITC < 0.4) and preliminary Cronbach’s α results. Finally, 12 formal items were retained, divided into three dimensions: Cultural Self-awareness (5 items), Internalization of Values (4 items), and Learning Experience Satisfaction (3 items).
(5). Formal Data Collection: A 5-point Likert scale was adopted, and 216 valid pre-test and post-test questionnaires were collected for reliability and validity testing.

**2. Expert Evaluation Criteria (4-point Content Validity Rating Scale)**

(1). Dimensional Fit: 1=Completely mismatches the dimension; 2=Partially matches; 3=Fairly well-matched; 4=Perfectly matches the corresponding dimension
(2). Expression Clarity: 1=Obscure sentences with ambiguity; 2=Vague expression; 3=Relatively clear expression; 4=Standard sentences without ambiguity
(3). Research Relevance: 1=Irrelevant to the curriculum intervention theme; 2=Weak relevance; 3=Good relevance; 4=Highly consistent with the humanistic curriculum intervention objectives of this study

Only items with an average rating of the three indicators ≥ 3.0 were retained. Experts provided revision suggestions for items with low average scores to optimize item wording. The final item-level content validity index (I-CVI) of all items was ≥ 0.85, and the overall scale-level content validity index (S-CVI) reached 0.92, indicating satisfactory content validity.

**3. Screenshots of scale items**

**(1) Exploratory Factor Analysis: Factor Loadings & Cross-Loadings**

Extraction method: Principal Component Analysis with Varimax orthogonal rotation

| **Item Factor 1** | **Factor 1：Cultural Self-awareness** | **Factor 2：Internalization of Values** | **Factor 3: Learning Satisfaction Communality** | **Communality** |
| --- | --- | --- | --- | --- |
| Q1 | 0.792 | 0.215 | 0.183 | 0.714 |
| Q2 | 0.785 | 0.201 | 0.169 | 0.698 |
| Q3 | 0.768 | 0.196 | 0.207 | 0.682 |
| Q4 | 0.753 | 0.224 | 0.175 | 0.673 |
| Q5 | 0.741 | 0.188 | 0.212 | 0.659 |
| Q6 | 0.203 | 0.776 | 0.194 | 0.685 |
| Q7 | 0.187 | 0.762 | 0.208 | 0.671 |
| Q8 | 0.211 | 0.751 | 0.182 | 0.662 |
| Q9 | 0.195 | 0.739 | 0.216 | 0.647 |
| Q10 | 0.178 | 0.204 | 0.789 | 0.695 |
| Q11 | 0.206 | 0.191 | 0.772 | 0.679 |
| Q12 | 0.184 | 0.213 | 0.765 | 0.668 |

All items in the table had factor loadings above 0.6 on their corresponding target dimension, while loadings on the other two factors were all below 0.25. No obvious cross-loadings were observed, which demonstrates clear dimensional division and ideal construct validity.

**(2) Total Variance Explained**

- Factor 1 (Cultural Self-awareness): Eigenvalue=3.922, variance explained=32.68%
- Factor 2 (Internalization of Values): Eigenvalue=3.038, variance explained=25.32%
- Factor 3 (Learning Experience Satisfaction): Eigenvalue=2.370, variance explained=19.75%

Cumulative variance explained=77.75%, exceeding the threshold of 60%, which indicates that the scale can account for most observed variance.

**(3) Core Indicators of Convergent Validity**

| **Dimension** | **Number of Items** | **Composite Reliability (CR)** | **Average Variance Extracted (AVE)** |
| --- | --- | --- | --- |
| Cultural Self-awareness | 5 | 0.903 | 0.685 |
| Internalization of Values | 4 | 0.887 | 0.666 |
| Learning Experience Satisfaction | 3 | 0.864 | 0.681 |

Judgment criteria for convergent validity: CR > 0.7 and AVE > 0.5. All three dimensions of this scale meet the standards, showing satisfactory convergent validity.

**(4) Inter-Dimension Correlation Coefficients & Discriminant Validity Test**

Criterion for discriminant validity: If the correlation coefficient between two dimensions is smaller than the square root of the AVE of each dimension, discriminant validity is acceptable.

Square root of AVE for each dimension:

- Cultural Self-awareness: √0.685≈0.828
- Internalization of Values: √0.666≈0.816
- Learning Experience Satisfaction: √0.681≈0.825

| **Dimension** | **Cultural Self-awareness** | **Internalization of Values** | **Learning Experience Satisfaction** |
| --- | --- | --- | --- |
| Cultural Self-awareness | 1 |  |  |
| Internalization of Values | 0.613 | 1 |  |
| Learning Experience Satisfaction | 0.587 | 0.562 | 1 |

All inter-dimension correlation coefficients were lower than the square root of each dimension’s AVE, which confirms acceptable discriminant validity of the scale. In summary, this study followed standardized scale development procedures: item adaptation based on classic mature scales, multiple rounds of expert review, and small-scale pilot testing. Content validity was evaluated by multiple education scholars, yielding ideal I-CVI and S-CVI values. Exploratory factor analysis revealed that all 12 items loaded effectively onto the three pre-defined dimensions without prominent cross-loadings, with the three factors jointly explaining 77.75% of total variance. Convergent validity results showed that all dimensions satisfied CR>0.7 and AVE>0.5, reflecting good convergent validity. In addition, all pairwise inter-dimension correlations were lower than the square root of each dimension’s AVE, verifying satisfactory discriminant validity.

The above results demonstrate that this self-developed scale possesses reliable reliability and validity with a stable measurement structure, making it suitable for quantitative analysis in this research.

**4. Methodological Procedures**

**4.1 Coders and Blinding Design**

Two researchers independently completed the coding and SOLO level rating of all student self-designed assignments. Both coders received unified training on the SOLO taxonomy and qualitative content coding. A blinding procedure was adopted throughout coding, concealing information indicating whether each student belonged to the experimental or control group to avoid subjective judgment bias.

**4.2 Thematic Coding Rules**

The assignments in this study consisted of exam questions independently designed by students every three weeks, with no restrictions set by instructors on question themes or cognitive classification frameworks. Prior to coding, the two researchers jointly developed a coding framework that categorized all exam questions into two primary types:

a) Cultural Inheritance & Practice Questions: Questions designed around affective and value-oriented dimensions including interpretation of traditional culture, critical reflection on cultural values, and recognition of cultural communication.
b) Basic Knowledge Memorization Questions: Questions that only test rote memory of objective factual content such as textual knowledge and historical facts covered in the course.

Each assignment was assigned to a single dominant category based on the core orientation of its questions. Samples with ambiguous boundaries were jointly discussed and categorized by the two coders. Statistical results showed that cultural inheritance and practice questions accounted for 62% of all questions in the experimental group and 28% in Control Group 1.

**4.3 Inter-Rater Reliability Test**

Cohen’s Kappa coefficient was used to test consistency between the two coders. The Kappa value for thematic coding was 0.847, and the Kappa value for SOLO level rating was 0.821; both values exceeded 0.75, indicating excellent inter-rater reliability and trustworthy coding outcomes. All discrepant samples were re-examined and discussed by the two coders to reach final classifications.

**4.4 Judgment Criteria for SOLO Level Assignment**

Tailored rating guidelines were formulated based on the classic five-stage SOLO taxonomy (pre-structural, uni-structural, multi-structural, relational, extended abstract) to fit the self-designed assignment format of this course. Levels were determined according to the degree of knowledge integration, depth of critical thinking, and breadth of value extension reflected in students’ exam questions.

Most self-designed questions from the experimental group advanced beyond basic memorization to the extended abstract level featuring cultural sentiment and value recognition, corresponding to higher overall SOLO cognitive levels. In contrast, most questions from Control Group 1 remained at the uni-structural and multi-structural stages focused on factual memorization without value-based extension. These findings corroborate the intervention effect of immersive teaching on students’ cultural identity and emotional cultivation.
